# Inhibitory Effect of Metalloproteinase Inhibitors on Skin Cell Inflammation Induced by Jellyfish *Nemopilema nomurai* Nematocyst Venom

**DOI:** 10.3390/toxins11030156

**Published:** 2019-03-10

**Authors:** Aoyu Li, Huahua Yu, Rongfeng Li, Song Liu, Ronge Xing, Pengcheng Li

**Affiliations:** 1 Key Laboratory of Experimental Marine Biology, Institute of Oceanology, Chinese Academy of Sciences, Qingdao 266071, China; liaoyu16@mails.ucas.ac.cn (A.L.); rongfengli@qdio.ac.cn (R.L.); sliu@qdio.ac.cn (S.L.); xingronge@qdio.ac.cn (R.X.); 2 Laboratory of Marine Drugs and Biological Products, Qingdao National Laboratory for Marine Science and Technology, Qingdao 266237, China; 3 Center for Ocean Mega-Science, Chinese Academy of Sciences, Qingdao 266071, China; 4 College of life sciences, University of Chinese Academy of Sciences, Beijing 100049, China

**Keywords:** *Nemopilema nomurai* nematocyst venom (NnNV), jellyfish dermatitis, matrix metalloproteinases (MMPs), inflammatory factors, skin cells

## Abstract

Jellyfish envenomations result in extensive dermatological symptoms, clinically named as jellyfish dermatitis, which can seriously affect the daily activities and physical health of people. Inflammatory response accompanies the whole process of jellyfish dermatitis and the complexity of jellyfish venom components makes it difficult to treat jellyfish dermatitis symptoms effectively. Moreover, inhibiting inflammation is essential for the treatment of jellyfish stings and exploring the main components of jellyfish venom that cause inflammation is an urgent research area. In this study, the inhibitory effects of matrix metalloproteinase (MMP) inhibitors for venom-induced inflammation were explored at a cellular level. The expression of the three inflammatory factors, IL-6, TNF-α and MCP-1 in two skin cell lines, human keratinocyte cells (HaCaT) and human embryonic skin fibroblasts cells (CCC-ESF-1), at the cellular level, after treatment with the inhibitors of jellyfish *Nemopilema nomurai* (*N. nomurai*) nematocyst venom (NnNV-I), were determined. The results showed that inhibitors of MMP can significantly reduce the toxic effects of jellyfish *Nemopilema nomurai* nematocyst venom (NnNV) to skin cells. The expression levels of the three inflammatory factors IL-6, MCP-1, and TNF-α in the cells were also significantly decreased, indicating that MMPs in jellyfish venom are probably vital factors leading to jellyfish dermatitis. This study is beneficial in the prevention and treatment of jellyfish stings.

## 1. Introduction

Jellyfish populations have sharply increased in recent years, contributing to a rapid soar in jellyfish injuries, which have seriously affected daily activities, such as coastal tourism, water sport activities, fishing, and soldier training, and have even caused death [1,2,3]. It was estimated that about 150 million people were stung by jellyfish annually according to the National Institutes of Health (NIH) [4]. The jellyfish *Nemopilema nomurai* (*N. nomurai*) is the main species of jellyfish which blooms in China, Korea, and Japan, and it is considered very dangerous to human beings. Once human beings are touched by *N. nomurai*, the venom in their nematocysts will discharge and inject into the skin, which results in jellyfish stings. Jellyfish stings cause a wide spectrum of symptoms in humans, ranging from mild pain and pruritis to excruciating pain with necrosis and scarring. Dermatitis is the main symptom and most victims show local cutaneous manifestations characterized by acute pain and inflammatory reactions [5]. The severity of a jellyfish stings depends on the dosage of venom penetrated into victims, composition and biological activity of the venom, the area stung, and the baseline health of the victims. It is undeniable that jellyfish stings are a tricky problem that needs serious attention [6].

Jellyfish dermatitis is the most probable symptom after jellyfish stings, and jellyfish venom is the cause of jellyfish envenomation. Considering the harm of jellyfish envenomation to humans, much research has been carried out to prevent and treat jellyfish stings or to reduce the toxic effects of jellyfish venom. Due to the diversity and complexity of jellyfish venom components, effective treatment of jellyfish stings is very difficult. Up to now, the treatment of jellyfish dermatitis is mainly based on the treatment of other dermatitis [7]; furthermore, acetic acid, ethanol, ammonia, and sodium bicarbonate are used for the first aid of jellyfish stings, but this has been controversial as these chemical reagents might stimulate the release of nematocysts and make the envenomations worse [8,9], and so the effectiveness of these methods is doubtful. Inflammatory symptoms of redness, swelling, and itching accompany the whole process of jellyfish stings, and so inhibiting inflammation is very important for the treatment of jellyfish stings. In addition, it is urgent to explore the main components of jellyfish venom which cause inflammation. The main component of jellyfish venom is protein. The molecular weight of NnNV is widely distributed, ranging from ~18.0 to ~235.7 kDa [10]. Proteomics and transcriptome results indicate that the jellyfish venom contains MMPs, phospholipase A2, protease inhibitors, potassium channel inhibitors, hemolysins, and other components [11]. Among them, MMPs account for an important proportion in the components. Various enzyme components in animal venom play an important role in the handling and digestion of prey, and MMPs of venom and are thought to induce a variety of toxicological effects, including wound bleeding, edema, and necrosis [12]. In addition, the MMPs in snake venom can damage the integrity of capillaries [13]. Then, skin damage and inflammation will also occur [14]. In view of the important role of MMPs in other toxins, we postulate that MMPs in jellyfish venom might be one of the most important components responsible for jellyfish dermatitis.

The complex and rich composition of venom, and the unstable and easily deactivated features make it difficult to obtain pure components and, up to now, MMPs have not been isolated and purified from jellyfish crude venom. In the process of purification, the activity of the venom will be partially lost, so we take jellyfish crude venom as the research object. In addition, jellyfish dermatitis caused by jellyfish stings may be the result of the interaction of various enzymes in jellyfish venom. Therefore, we use MMP inhibitors to react with jellyfish crude venom, in order to inhibit the MMPs in jellyfish venom and explore whether the MMPs in jellyfish venom are related to jellyfish dermatitis. Ethylenediaminetetraacetic acid (EDTA) chelates the MMPss active center, inducing conformational change of the metalloproteinase, leading to theirs inactivation. Batimastat (BMT) is a hydroxamate peptidomimetic that binds to zinc ions in MMPs and specifically inhibits enzyme activity. BMT has a good inhibitory effect on the toxicity of snake venom [15] and it can inhibit the hemolytic [16] and enzymatic [10] activity of jellyfish venom. Moreover, BMT can work against the hemorrhagic injuries resulting from jellyfish venom [17]. EDTA also has a good effect of inhibiting the hemolytic activity of jellyfish venom [16]. Hence, we chose BMT and EDTA as the subjects of this study.

Jellyfish dermatitis has rarely been studied from the perspective of inflammatory factors, even though jellyfish stings can cause severe skin injuries, such as redness, swelling, and ulceration. Therefore, it is very important and necessary to investigate the expression level of cellular inflammatory factors for the treatment of jellyfish dermatitis.

In this study, the effect of NnNV on the expression of the three inflammatory cytokines of IL-6, MCP-1, and TNF-α were determined, at the cellular level, using two typical skin cells, HaCaT and CCC-ESF-1, as carriers. The effects of the MMP inhibitors BMT and EDTA on NnNV were also investigated to ascertain the metalloproteinase responsible for jellyfish dermatitis and MMP inhibitors that have potential for utilization as a treatment of jellyfish dermatitis.

## 2. Results

### 2.1. Effect of NnNV and NnNV-I on the Viability of HaCaT and CCC-ESF-1

**Figure 1:**
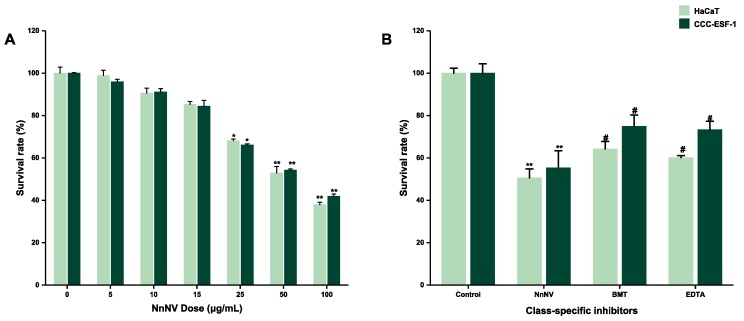
(**A**) Effect of *Nemopilema nomurai* nematocyst venom (NnNV) on the viability of HaCaT and CCC-ESF-1 cells. HaCaT and CCC-ESF-1 cells were treated with various concentrations of NnNV for 24 h and cell viability was measured using the MTT assay. (**B**) Effect of inhibitors of NnNV (NnNV-I) on the viability of HaCaT and CCC-ESF-1 cell. HaCaT and CCC-ESF-1 cells were treated with the mixture of NnNV (50 μg/mL, final concentration) and BMT (0.5 μM, final concentration) or EDTA (20 μM, final concentration) for 24 h and cell viability was measured with the MTT assay. * *p* < 0.05, ** *p* < 0.005, and *** *p* < 0.001 vs. Control (to verify the effect of NnNV on cell viability), ^#^ *p* < 0.05, ^##^ *p* < 0.005, and ^###^ *p* < 0.001 vs. NnNV (to verify that MMP inhibitors can reduce the inhibition rate of NnNV on cells).

As shown in Figure 1A, when the HaCaT and CCC-ESF-1 cells were treated with NnNV for 24 h, the viability of these two kinds of cells was significantly decreased in a dose-dependent manner. We wanted conditions that would damage cells as much as possible, to highlight the inhibitory effect of MMP inhibitors on venom, but not such that the number of cells in the NnNV group would be too small to collect, due to cell death. Therefore, based on the results of Figure 1A, we selected 50 μg/mL NnNV mixed with either BMT or EDTA for subsequent experiments, and the survival rate of the two cell lines was approximately 50% in this concentration. The results (Figure 1B) showed that the survival rate of the two skin cell lines was significantly increased after the addition of NnNV-I (compared with NnNV group), and these two inhibitors had no cytotoxicity at this concentration. The inhibition rate of cell survival at 50 μg/mL NnNV was about 50%. When NnNV mixed with either BMT or EDTA was applied, the survival rate of the cells reached about 70%. The cell survival rate was increased by about 20% with the addition of MMP inhibitors. These results indicate that the MMP inhibitors significantly reduced the toxicity of NnNV to cells. In addition, the experimental results showed that there was no significant difference between the two MMP inhibitors on the inhibition of NnNV.

### 2.2. Effect of NnNV on the Gene Expression of IL-6, MCP-1, and TNF-α in HaCaT and CCC-ESF-1 at Different Times

**Figure 2:**
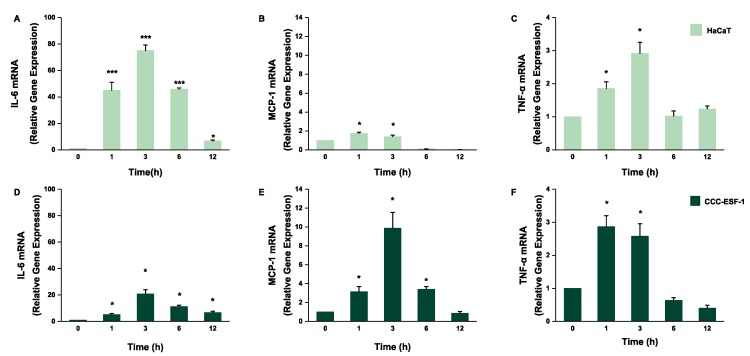
Effect of NnNV on the gene expression of *IL-6*, *MCP-1*, and *TNF-α* in HaCaT and CCC-ESF-1 cells at different times. (**A**–**C**) HaCaT and (**D**–**F**) CCC-ESF-1 were treated with 10 μg/mL NnNV for various times (0, 1, 3, 6, and 12 h). * *p* < 0.05, ** *p* < 0.005, and *** *p* < 0.001 vs. Control.

Based on the results of Figure 1A, in order to ensure that there are enough live cells, 10 μg/mL NnNV was selected. When assessing 10 μg/mL NnNV treatment of the two kinds of cells over different time periods, the content of the three inflammatory factors in cells were sharply increased in a short period of time and reached the highest level in 3 h, then, the expression of inflammatory factors decreased and finally stabilized (Figure 2), indicating that NnNV probably induced acute inflammation.

### 2.3. Effect of NnNV and NnNV-I on the Gene Expression of IL-6, MCP-1 and TNF-α in HaCaT and CCC-ESF-1

**Figure 3:**
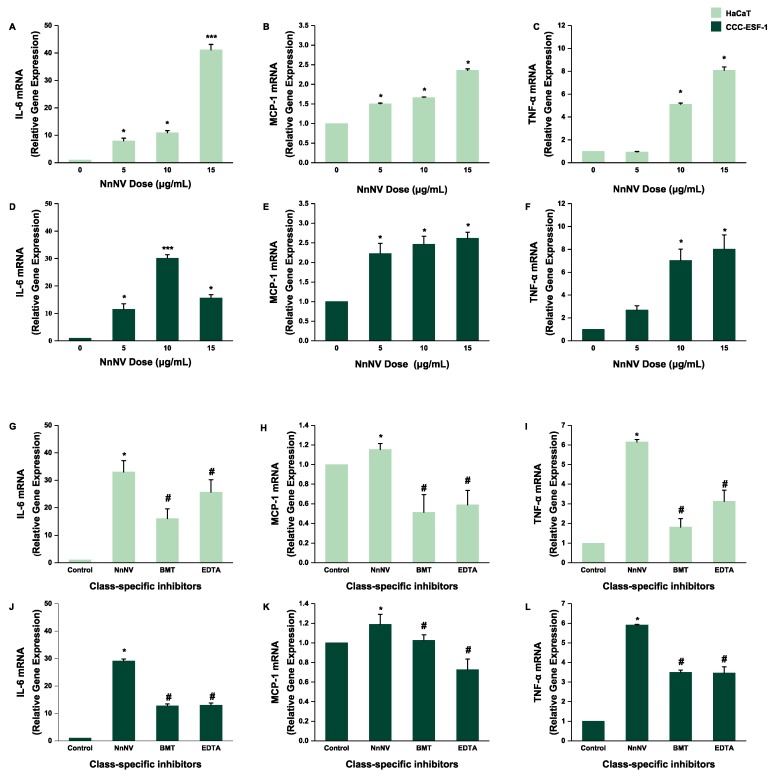
Effect of NnNV and NnNV-I on the gene expression of *IL-6*, *MCP-1* and *TNF-α* in HaCaT and CCC-ESF-1 cells. (**A**–**C**) HaCaT and (**D**–**F**) CCC-ESF-1 were treated with various concentrations of NnNV for 24 h. (**G**–**I**) HaCaT and (**J**–**L**) CCC-ESF-1 were treated with various NnNV-I for 24 h. * *p* < 0.05, ** *p* < 0.005 and *** *p* < 0.001 vs. Control (to verify that NnNV can promote mRNA expression of the inflammatory factors in cells), ^#^ *p* < 0.05, ^##^ *p* < 0.005 and ^###^ *p* < 0.001 vs. NnNV (to verify that MMP inhibitors can reduce mRNA expression of the inflammatory factors of NnNV in cells).

According to Figure 1A, in order to ensure that there were enough live cells, 5, 10 and 15 μg/mL NnNV were selected. When the two kinds of cells were treated with different concentrations of NnNV, the mRNA expression of *TNF-α*, *IL-6* and *MCP-1* in the cells increased significantly, and the highest gene expression of *IL-6* was about 30 to 40 times that of the control group, and the gene expression of *TNF-α* in both cells was about 8 times that of the control group. However, the mRNA expression level of *MCP-1* was different from that of the other two inflammatory factors, and the highest expression was only about 2.5 times that of the control group. The mRNA expression of *MCP-1* in CCC-ESF-1 cells increased slightly, but there was no significant change with the increase of NnNV concentration (Figure 3). As can be seen from Figure 3, when BMT and EDTA were added to the venom, the gene expression levels of the three inflammatory factors were significantly lower than that of the NnNV group.

### 2.4. Effect of NnNV and NnNV-I on the Protein Expression of IL-6, MCP-1 and TNF-α in HaCaT and CCC-ESF-1

**Figure 4:**
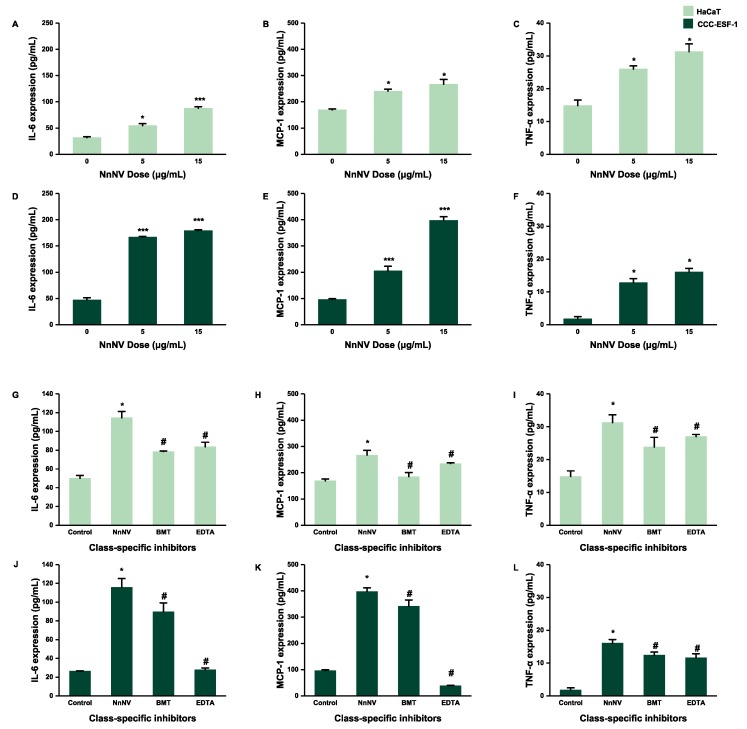
Effect of NnNV and NnNV-I on the cell protein expression of IL-6, MCP-1 and TNF-α on HaCaT and CCC-ESF-1 cells. (**A**–**C**) HaCaT and (**D**–**F**) CCC-ESF-1 were treated with various concentrations of NnNV for 24 h. (**G**–**I**) HaCaT and (**J**–**L**) CCC-ESF-1 were treated with various NnNV-I for 24 h. * *p* < 0.05, ** *p* < 0.005, and *** *p* < 0.001 vs. Control (to verify that NnNV can promote the protein expression of inflammatory factors in cells). ^#^ *p* < 0.05, ^##^ *p* < 0.005, and ^###^ *p* < 0.001 vs. NnNV (to verify that MMP inhibitors can reduce the protein expression of inflammatory factors of NnNV in cells).

The protein expression of three inflammatory factors in cells shows a trend of NnNV-dependent increase (Figure 4A–F). The protein expression of MCP-1 is the highest. Perhaps the release rate of MCP-1 protein may be faster than that of the other two among the three inflammatory factors.

As shown in the Figure 4G–L, the protein content of intracellular inflammatory factors in NnNV-I group was significantly lower than that of the NnNV group. MMP inhibitors of BMT and EDTA had significant inhibitory effects on the protein expression of three inflammatory factors of IL-6, MCP-1 and TNF-α in cells as induced by NnNV. The inhibitory effects of BMT and EDTA on NnNV were similar in HaCaT (Figure 4G–I). In CCC-ESF-1 cells, the protein expression of EDTA-treated NnNV was significantly lower than that of BMT-treated group (Figure 4J,K), indicating that the inhibitory effect of EDTA on NnNV was higher than that of BMT-treated group. The contents of intracellular inflammatory factors of treatment groups were significantly higher than that of the control group (Figure 3 and Figure 4). With the increase in concentration of NnNV, the content of inflammatory factors also had an increasing trend. In summary, the data show that the NnNV can promote the expression of inflammatory factors significantly, and the MMP inhibitors of EDTA and BMT can also significantly inhibit the inflammatory effects of NnNV.

## 3. Discussion

Skin is a complex network that regulates pro-inflammatory and anti-inflammatory effects, and many skin diseases are associated with inflammation [18,19]. Skin tissue can be divided into the epidermis and dermis. HaCaT cells are epidermis cells, playing an important role in skin protection, while CCC-ESF-1 cells are dermis cells and play a crucial role in the repair of skin tissue. During jellyfish stings, jellyfish venom first contacts with the epidermis of skin tissues. After passing through the epidermis, jellyfish venom reaches the dermis and finally reaches the subcutaneous tissues and muscles, which lead to different toxic symptoms. Dermatitis is the most direct symptom of venom to skin tissues. Therefore, we chose HaCaT and CCC-ESF-1 cells to explore the effect of MMPs on the inflammation of NnNV at the cellular level. The present study focused on investigating the effects of NnNV on inflammatory factors in the two skin cells and the effect of MMPs on jellyfish dermatitis at the cellular level. TNF-α is the earliest and most important inflammatory mediator, which is believed to play important roles in the process of inflammations, such as increasing the permeability of vascular endothelial cells, activating neutrophils and lymphocytes, and promoting the synthesis and release of other cytokines. Moreover, it plays an important role in apoptosis and immune response [20]. IL-6 is a multipotent cytokine that plays a vital regulatory role in inflammation [21] and is an accelerator for inflammatory reactions. IL-6 expression levels are elevated in some inflammatory reactions, such as infection, skin trauma, rheumatoid arthritis, and systemic lupus erythematosus [22]. MCP-1 is known as a chemokine; when the human body is infected by foreign bodies, leukocytes tend to aggregate to the infected site. Controlling cell migration during normal repair or development, excessive production can also aggravate the progression of inflammation. These three inflammatory factors are used as indicators of inflammation to explore the inflammatory effects of other toxins, for example, IL-6 and TNF-α were used as indicators of inflammation to explore the inflammatory effects of snake venom [23]. MCP-1 and IL-6 were used to explore the inflammatory effects of spider venom [24]. Upregulation of proinflammatory cytokines contributes to enhanced monocyte adhesiveness and infiltration into the skin during the pathogenesis of various inflammatory skin diseases, such as atopic dermatitis. However, any overproduction of these factors can be responsible for harmful inflammatory reactions.

The results indicate that NnNV could stimulate cells to express a large number of inflammatory factors *IL-6*, *MCP-1*, and *TNF-α* in a short period of time (Figure 2) and, in the process of actual stings, the stung body would show different inflammatory reactions in a short time. The production of this symptom may be associated with the phenomenon of jellyfish venom promoting the production of inflammatory factors in cells in a short time. Furthermore, the gene expression levels of the three inflammatory factors *IL-6*, *MCP-*1, and *TNF-α* were significantly increased. In addition, the gene expression levels of all inflammatory factors increased in a dose-dependent manner (Figure 3), indicating that the action of NnNV on the skin cells may lead to the occurrence of jellyfish dermatitis. The protein expression of inflammatory factors in cells showed a significant upward trend with the increase of NnNV concentration, and when the metalloproteinase inhibitors BMT or EDTA acted on NnNV, the protein expression level of the inflammatory factors in cells was significantly lower than that of the NnNV group; this was the same as the trend of gene expression level of the inflammatory factors in cells. The protein expression level in cells is an absolute quantitative marker of inflammation, while the gene expression level is a quantitative change relative to the control group. We measured both the gene expression and protein expression levels of inflammatory factors in order to better verify that NnNV can promote the production of inflammatory factors, and MMP inhibitors BMT or EDTA can reduce the inflammatory effects of venom.

In recent years, some studies have shown that the enzyme components in toxins play an important role in the poisoning of toxic organisms, wounds, bleeding, and festering caused by the stings or bites of poisonous organisms such as centipedes, snakes, and ticks [25,26]. Inhibitors of MMPs have been used to prevent secondary infections or inhibit the hemorrhagic activities of snake venoms. MMPs were detected in the venom of jellyfish *Stomolophus meleagris* [11], *Chironex fleckeri* [27], and *Nemopilema nomurai* [28]. Although it has been reported that there are abundant proteases in jellyfish venom [29], and that MMPs play an important role in the lethal toxicity [30], hemolytic toxicity [16], cytotoxicity [28], and cardiotoxicity [17] of jellyfish venom, no mention has been made of the effect of MMPs on jellyfish dermatitis. Our results indicate that MMPs in jellyfish venom probably play an important role in the process of jellyfish dermatitis caused by jellyfish stings. Dossena pointed out that MMPs can be regarded as novel therapeutic targets in scyphozoan envenomation [31]. This view suggested that MMPs may play an important role in jellyfish stings, which is consistent with the results of this study.

Although MMP inhibitors do not completely inhibit the toxicity of NnNV, it is undeniable that they significantly reduce the content of inflammatory factors in cells after the toxic effect and increase the cell survival rate. Therefore, the MMP inhibitors may be effective in the treatment of jellyfish dermatitis.

In conclusion, venom from the jellyfish *N. nomurai* enhances the expression of inflammatory factors IL-6, TNF-α, and MCP-1 in two typical skin cells. The MMP inhibitors, EDTA and BMT, reduced the expression of these inflammatory factors. These results indicate that MMPs in the jellyfish venom are probably important components responsible for jellyfish dermatitis, and that inhibitors of MMP are promising candidates for the treatment of jellyfish dermatitis. This study reveals the characteristics of inflammatory factors of jellyfish dermatitis for the first time, which provides references for the clinical treatment of jellyfish dermatitis. The mechanism of MMPs on jellyfish dermatitis, the feasibility and accuracy of MMPs as a target for jellyfish dermatitis, and the effects of MMP inhibitors on inflammation, in vivo, will be further studied. As for the appropriate timeframe to apply MMP inhibitors for jellyfish dermatitis treatment, this needs to be determined through animal experiments. However, according to the results of this study and the clinical reaction of jellyfish stings, jellyfish dermatitis is an acute reaction, and so the treatment time should be as soon as possible, to reduce the toxicity of venom and ease symptoms.

## 4. Materials and Methods

### 4.1. Jellyfish Collection

Jellyfish specimens of *N. nomurai* were collected from the Laoshan Bay in Qingdao, China, in August 2017. The jellyfish tentacles were immediately and manually excised from live specimens using scissors, and stored at −80 °C for nematocyst isolation and venom extraction. The animal experimental protocols were approved by the Institutional Committee for the Care and Use of Animals of Institute of Oceanology, CAS, ethical approval number-jellyfish used for experiments is KLEMB—LL—2017—006.

### 4.2. Nematocyst Isolation and Venom Extraction

Nematocysts were isolated from *N. nomurai* tentacles according to the methods described by Bloom [32] and Carrett [33]. Briefly, the frozen tentacles were thawed in filtered seawater at 4 °C for autolysis. The seawater was changed every day until the majority of the visible tissue debris was dissolved. Meanwhile, a glass bar was used to stir the mixtures thoroughly. After autolysis, the mixtures were filtered through a 100-mesh plankton net to remove the tissue debris. The filtrate was then centrifuged at 3000× *g* for 15 min at 4 °C, and the nematocysts were obtained by collecting the resultant sediments. The nematocysts were repeatedly washed three times with cold venom extraction buffer (VEB, 20 mM PO_4_^3−^, 150 mM NaCl, pH 7.4), and further centrifuged at 10,000× *g* for 15 min at 4 °C. Then, the nematocysts were immediately used for venom extraction or, alternatively, frozen at −80 °C until use.

Venom was extracted from the nematocysts, similar to the methods described by Rongfeng Li [34]. Briefly, cold VEB was used to suspend the nematocysts and the nematocysts were extracted by an Ultraturrax (JY92-II, Scientz, Zhejiang, China) at 400 W for 90 cycles, with each cycle consisting of 10 s sonication and a 15 s interval rest on ice. The venom extracts were collected and centrifuged at 12,000× *g* for 15 min at 4 °C. The resultant supernatants were the nematocyst venom of the jellyfish *N. nomurai*, and we marked the nematocyst venom of jellyfish *N. nomurai* as NnNV, for convenience.

### 4.3. Cell Culture

Human keratinocyte cells (HaCaT) and human embryonic skin fibroblasts cells (CCC-ESF-1) were purchased from China Infrastructure of Cell Line Resource. The HaCaT cells were cultured in MEM-EBSS (Hyclone, Waltham, MA, USA) supplemented with 10% fetal bovine serum (FBS, Gibco, Carlsbad, CA, USA). CCC-ESF-1 cells were cultured in high glucose DMEM (Hyclone, Waltham, MA, USA) supplemented with 20% fetal bovine serum (FBS, Gibco, Carlsbad, CA, USA), and 100 U/mL penicillin and 100 μg/mL streptomycin were added to both types of culture media. The cells were incubated in an incubator, with atmospheric conditions of 37 °C, 95% air, 5% CO_2_, and 100% humidity.

### 4.4. Cell Viability Assay

Cell viability was determined using the MTT assay. The MTT assay is a method for detecting cell survival and growth. The detection principle is that succinate dehydrogenase in living cell mitochondria can reduce exogenous MTT to water-insoluble blue-purple crystalline formazan and deposit in cells, while dead cells do not have this function. The two kinds of cells were plated in 96-well culture plates at a density of 1 × 10^6^ cells/mL. Three duplicates were set up for each set of experiments. After incubation for 24 h, cell groups were treated with NnNV (5, 10, 15, 25, 50 and 100 μg/mL) and NnNV-I for 24 h, respectively. Since there is no study on the skin toxicity of BMT and EDTA to jellyfish venom, based on the studies of the effects of BMT and EDTA on the hemolytic activity of NnNV [16], the approximate concentration of the two inhibitors were obtained, and preliminary experiments were carried out to determine the final concentration of BMT and EDTA (Figure S1). Venom solutions were incubated for 30 min with BMT to investigate the effect of BMT on the pathological effects induced by *B. asper* venom [15], and the effect of hemolytic activity of NnNV was determined after the reaction of NnNV with BMT or EDTA for 30 min [16]. Hence, NnNV was reacted with BMT or EDTA for 30 min in follow-up experiments. BMT (0.5 μM, final concentration) or EDTA (20 μM, final concentration) were pretreated with NnNV (50 μg/mL, final concentration) for 30 min [15,16], and these mixtures were named as NnNV-I.

Then, the media was removed and MTT (0.5 mg/mL) was added to each well for 4 h. Formazan crystals from MTT reduction were dissolved in stopping buffer. The absorbance value at 490 nm was measured using an Infinite M100 plate reader (Tecan Group Ltd., Männedorf, Switzerland).

### 4.5. RNA Isolation and Quantitative Real Time PCR (qRT-PCR)

HaCaT and CCC-ESF-1 cells (1.0 × 10^6^ cells/mL) were treated with 10 μg/mL NnNV for different times (0, 1, 3, 6 and 12 h). Then, total RNA was isolated for subsequent experiments.

Total RNA was isolated from HaCaT or CCC-ESF-1 cells, after appropriate treatment, using the RNeasy RNA isolation kit (Omega Bio-Tek, Norcross, GA, USA) according to the manufacturer’s guidelines. For each qRT-PCR, 0.8 μg of total RNA was used. Total RNA was converted to cDNA with an iScript cDNA Synthesis kit (TaKaRa Bio inc, Kusatsu, Japan). Three duplicates were set up for each set of experiments. Then, qRT-PCR was performed using SYBR Green Master Mix (TaKaRa Bio inc) according to the manufacturer’s instructions.

The cDNA amplification of a specific sequence of human *IL-6*, *MCP-1*, *TNF-α*, and *β-actin* were performed by qRT-PCR using the primer sequences in Table 1. Gene expression was calculated using the delta CT method with *β-actin* as reference gene according to Pfaffl [35]. Briefly, the gene *β-actin* was used as the internal control. The relative mRNA expression of *IL-6*, *MCP-1*, and *TNF-α* was calculated by normalizing to *β-actin*.

HaCaT and CCC-ESF-1 cells (1.0 × 10^6^ cells/mL) were treated with different concentrations of NnNV (0, 5, 10, and 15 μg/mL) and NnNV-I for 24h. Then, the total RNA was isolated for subsequent experiments according to the method mentioned above.

### 4.6. ELISA Detection of Cytokines

For cellular supernatant detection, HaCaT and CCC-ESF-1 cells (1.0 × 10^6^ cells/mL) were cultured in a 96-well plate. Three duplicates were set up for each set of experiments. After incubation for 24 h, cell groups were separately treated with NnNV (0, 5, 15 μg/mL) and NnNV-I for 24 h. Then, the cellular supernatant samples were collected and subjected to centrifugation at 1000× *g* (Thermo Fisher Scientific, Waltham, MA, USA) for 10 min and stored at −20 °C until use. The production of cytokines (IL-6, MCP-1, and TNF-α) in the supernatant was quantified with an ELISA kit (Abcam, Cambridge, UK).

### 4.7. Statistical Analysis

All values in the figures and text are expressed as mean values ± SEM. The significance of differences between the means of various experimental groups was analyzed by one-way ANOVA, followed by Tukey’s multiple comparisons test, which are built into GraphPad Prism v6.0 (GraphPad software, San Diego, CA, USA). * *p* < 0.05, ** *p* < 0.01, and *** *p* < 0.001 were considered statistically significant.

## Figures and Tables

**Table 1:** Primer sequences of *IL-6*, *MCP-1*, *TNF-α*, and *β-actin.*

Primer Pairs	
β-actin	F: 5′-GGCACCACACCTTCTACAATGAGC-3′
	R: 5′-GATAGCACAGCCTGGATAGCAACG-3′
IL-6	F: 5′-GTCCAGTTGCCTTCTCCC-3′
	R: 5′-GCCTCTTTGCTGCTTTCA-3′
MCP-1	F: 5′-CTTCTGTGCCTGCTGCTC-3′
	R: 5′-TGCTGCTGGTGATTCTTCT-3′
TNF-α	F: 5′-CGTGGAGCTGGCCGAGGAG-3′
	R: 5′-AGGAAGGAGAAGAGGCTGAGGAAC-3′

F: Forward primer, R: Reversed primer

## Supplementary Materials

The following are available online at https://www.mdpi.com/2072-6651/11/3/156/s1, Figure S1. Effects of different concentrations of BMT and EDTA on cell viability. (A) CCC-ESF-1 cells were treated with different concentrations of BMT. (B) CCC-ESF-1 cells were treated with different concentrations of EDTA.

## Abbreviations

NnNV	jellyfish *Nemopilema nomurai* nematocyst venom
NnNV-I	the mixtures of jellyfish *Nemopilema nomurai* nematocyst venom and matrix metalloproteinase inhibitors batimastat or ethylenediaminetetraacetic acid
BMT	batimastat
EDTA	ethylenediaminetetraacetic acid
PBS	phosphate-buffered saline
FBS	fetal bovine serum
MMP	matrix metalloproteinase
VEB	venom extraction buffer
