# Supplementary material for: Inhibitory Effect of Metalloproteinase Inhibitors on Skin Cell Inflammation Induced by Jellyfish Nemopilema nomurai Nematocyst Venom
Source: Toxins (Basel). 2019 Mar 10;11(3):156. doi: 10.3390/toxins11030156 (PMC6468808; doi:10.3390/toxins11030156)
Supplement: Supplementary file 1 [file toxins-11-00156-s001.pdf]

toxins

Supplementary Materials: Inhibitory Effect of Metalloproteinase Inhibitors on Skin Cells Inflammation Induced by Jellyfish *Nemopilema nomurai* (*N. nomurai*) Nematocyst Venom

Aoyu Li, Huahua Yu, Rongfeng Li, Song Liu, Ronge Xing and Pengcheng Li

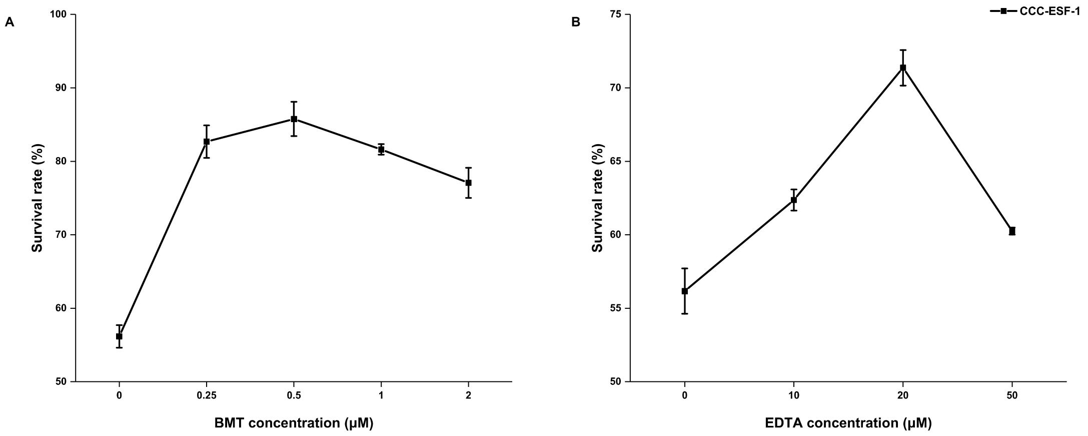

Figure S1. Effects of different concentrations of BMT and EDTA on cell viability. (**A**) CCC-ESF-1 cells were treated with different concentrations of BMT. (**B**) CCC-ESF-1 cells were treated with different concentrations of EDTA.
